# Complete remission in children and adolescents with type 1 diabetes mellitus—prevalence and factors

**DOI:** 10.1038/s41598-023-34037-7

**Published:** 2023-04-26

**Authors:** Kristina Podolakova, Lubomir Barak, Emilia Jancova, Simona Tarnokova, Ludmila Podracka, Zuzana Dobiasova, Martina Skopkova, Daniela Gasperikova, Juraj Stanik

**Affiliations:** 1 Department of Pediatrics, Medical Faculty of Comenius University and National Institute for Children’s Diseases, Limbova 1, 833 40 Bratislava, Slovakia; 2 Department of Laboratory Medicine, National Institute for Children’s Diseases, Limbova 1, 833 40 Bratislava, Slovakia; 3 Department of Metabolic Disorders, Institute of Experimental Endocrinology, Biomedical Research Center, Slovak Academy of Sciences, Dubravska Cesta 9, 845 05 Bratislava, Slovakia

**Keywords:** Genetic markers, Type 1 diabetes

## Abstract

Little is known about complete remission in Type 1 diabetes mellitus (T1D) with the discontinuance of insulin treatment for a period of time. In this retrospective study we analysed the frequency and factors of onset and duration of 1. remission and 2. complete remission in children and adolescents with T1D from the Children Diabetes Centre in Bratislava, Slovakia. A total of 529 individuals with T1D, aged < 19 years (8.5 ± 4.3 years) at diabetes onset were included in the study. Remission was defined by HbA1c < 7.0% (53 mmol/mol) and an insulin daily dose < 0.5 IU/kg (and 0 IU/kg for complete remission). Remission occurred in 210 (39.7%) participants, and 15 of them had complete remission (2.8% from all participants). We have identified a new independent factor of complete remission onset (higher C-peptide). Complete remitters had a longer duration of remission compared with other remitters and also differed in lower HbA1c levels. No association was seen with autoantibodies or genetic risk score for T1D. Thus, not only partial but also complete remission is influenced by factors pointing toward an early diagnosis of T1D, which is important for better patient outcome.

## Introduction

Remission in T1D, also known as the “honeymoon” period, is characterised by a decline of insulin doses and stable glycaemia shortly after initialisation of insulin treatment^1^. Almost 80% of children and adolescents show a decline of initial insulin requirements accompanied by an increase of endogenous insulin production, and many of them also have good glycaemic control despite mistakes in exercise and diet^1^. Remission seems to be a valuable positive factor for the further T1D course, as the preservation of B-cell function may decrease the risk of developing vascular complications and the risk of severe hypoglycaemia^1,2^.

Remission can be partial or complete. Partial remission is a frequent finding in people with newly diagnosed type 1 diabetes mellitus (T1D) and is defined by ISPAD as an insulin requirement of < 0.5 units per kg of body weight per day and HbA1c < 7% (53 mmol/mol)^1^**.** Alternatively, some studies use the definition for partial remission given by Mortensen et al. based on the insulin dose-adjusted HbA1c (IDAA1c), calculated as: HbA1c (%) + (4 × insulin dose (units per kilogram per 24 h). As a cut-off for partial remission they suggested an IDAA1c value of ≤ 9^3^. In the case of complete remission, no insulin treatment is needed for a period of time. Spontaneous complete remission is less common (mostly < 3% in children and adolescents with T1D)^4^, and studies describing children and adolescents with the complete remission in T1D are also rare^4,5^. However, both partial and complete remission in T1D do not have a very long duration and usually disappear within several weeks to years. Remission lasting longer than 3 years may signal a misdiagnosis for another type of diabetes mellitus (i.e. monogenic diabetes or type 2 diabetes)^1,6^.

Several clinical and metabolic factors have been found to have an impact on the occurrence and duration of partial remission by studies conducted in children and adolescents. These factors include age, duration of symptoms, ketoacidosis at diabetes onset, C-peptide, HbA1c levels, body mass index standard deviation score (BMI-SDS), sex and time to remission onset^4,7–12^. Factors of complete remission remain unclear due to the low frequency of the condition.

In our retrospective study we analysed the frequency and factors of the onset and duration of remission for 1. all remitters and 2. complete remitters in children and adolescents with newly diagnosed T1D. We also aimed to ascertain why some patients are able to achieve complete remission.

## Methods

### Patients

In this retrospective study, we included children and adolescents aged < 19 years with T1D diagnosed and followed-up in the Children Diabetes Centre at the Department of Paediatrics, Faculty of Medicine, Comenius University and National Institute of Children’s Diseases in Bratislava, Slovakia, in the years from 2010 to 2019. We excluded individuals with T1D who participated in clinical intervention trials, as well as individuals with other types of diabetes mellitus..

### Diabetes evaluation

Diagnosis of T1D was based on the current criteria of the International Society for Pediatric and Adolescent Diabetes (ISPAD)^13^. All included patients required ongoing insulin therapy (children with complete remission required insulin therapy after the end of complete remission), plus had at least one of the following features: diabetic ketoacidosis (DKA) at the onset of diabetes, detection of one or more T1D-associated antibodies (glutamic acid decarboxylase, protein tyrosine phosphatase, islet cell or insulin autoantibodies), or apparent decline of C-peptide serum levels (< 200 pmol/l) during the first three years after the T1D diagnosis.

#### Hospitalisation, diabetic ketoacidosis and start of insulin treatment

All children and adolescents with the newly diagnosed T1D included in the study were hospitalised for approximately 6–12 days. Patients with diabetic ketoacidosis were treated according to the ISPAD guidelines^14^. The severity of the DKA was defined as follows: severe DKA with pH < 7.10 or serum bicarbonate < 5 mmol/l; moderate DKA with pH 7.10–7.20, or serum bicarbonate 5–10 mmol/l; and mild DKA with pH 7.20–7.30 or serum bicarbonate 10–15 mmol/l^14^. Thereafter, all children and adolescents with T1D were set on insulin treatment with multiple daily insulin injections in an intensified insulin regime (combination of multiple injections of pre-prandial regular insulin or short acting insulin analogue with the NPH insulin or long acting insulin analogue). The insulin dose was titrated according to the glycaemia.

#### Recorded parameters

Age, sex, anthropometric data, glycaemia, pH, serum bicarbonate, ketonuria, HbA1c, fasting C-peptide levels, triglycerides and diabetes-associated autoantibodies (against glutamic acid decarboxylase, tyrosine phosphatase and insulin) were recorded at the time of the diabetes diagnosis. Insulin dose, height and weight were also recorded at the time of discharge from hospital and each 3 months in the follow-up.

A diagnosis of T1D associated autoimmune diseases was made on the basis of TSH and FT4 levels, thyroidal autoantibodies and ultrasound (Hashimoto thyroiditis), and based on anti-endomysial and anti-transglutaminase autoantibodies or intestinal biopsy (celiac disease).

### Remission

Remission was determined on the basis of the HbA1c values < 7% (53 mmol/mol) and the daily insulin requirement < 0.5 IU/kg/day (ISPAD Consensus Guidelines 2018)^1^ for partial remission, or no insulin requirement for complete remission. In the complete remitters, the insulin treatment was suspended when the insulin requirement fell to 0.5 IU/day, accompanied by most blood glucose measurements being in the target range (fasting 4–6 mmol/l, postprandial < 10 mmol/l) and frequency of hypoglycaemia (< 3.9 mmol/l) more than 1 episode/week.

For additional information on epidemiology of remission using more strict criterion of HbA1c (i.e. < 6% (48 mmol/mol)) look at supplemental material S-1.

### Biochemical analyses

Bicarbonate levels, pH and glucose were determined in capillary blood samples taken at the time of the patients being admitted to the Children Diabetes Centre in Bratislava. Serum lipids, C-peptide and autoantibodies levels were measured within 3 days after the patients had been admitted to the hospital. Fasting serum lipid levels were measured using standardised methods. C-peptide levels were determined using the electrochemiluminescence immunoassay method, pancreatic autoantibodies (against glutamic acid decarboxylase, tyrosine phosphatase and insulin) were detected using ELISA kits. Celiac autoantibodies (against transglutaminase and against endomysium IgA, IgG) were measured by indirect immunofluorescence assay. Thyroidal autoantibodies (against thyroid peroxidase and thyroglobulin) were determined by electrochemiluminescence immunoassay. HbA1c was evaluated from whole blood using the HPLC method and was initially measured within 3 days after the patient was admitted to the hospital and then every 3 months during the follow-up. All HbA1c values were transformed to Diabetes Control and Complications Trial (National Glycohemoglobin Standardisation Programme percentage) and International Federation of Clinical Chemistry values (http://www.ngsp.org/ifccngsp.asp).

### Genetic risk score for T1D and HLA antigens

A subset of 398 individuals from the cohort were available for genotyping. DNA was extracted from peripheral blood and was subjected to Illumina Infinium Global Screening beadchip GSA MD (Infinium Iselect 24 × 1 HTS Custom Beadchip Kit) provided by HuGe-F Erasmus University Medical Center, Rotterdam, Netherlands, as a service.

The top 10 single nucleotide polymorphisms (SNPs) with the highest discriminative power of the T1D GRS^16,17^ were used for the genetic risk score (GRS) computation according to Oram et al.^16^. The SNPs in samples that failed to be genotyped in sufficient quality (with a call score < 0.27) were assessed individually using KASP assay (LGC, Biosearch Technologies). The human leucocyte antigens (HLA) alleles were also separately evaluated beside the GRS, i.e. carriers of the DR3/DR4 haplotype with a very high risk, carriers of DR4/DR4 or DR3/DR3 haplotypes with a high risk, carriers of one DR3 or DR4 allele with a medium risk, and carriers of none of the risk alleles with a low genetic risk for T1D.

### Statistical analysis

Metric data were checked for normality using the Shapiro–Wilk test. Normally distributed data are expressed as the mean ± SD. Non-normally distributed data (i.e. duration of remission, triglycerides and C-peptide levels) are expressed as the median and interquartile range (25th –75th percentiles). Non-normally distributed data were logarithmically transformed prior to further statistical analyses. Confidence intervals for percentages in binary data were calculated using the Wilson/Brown method. Differences between the two groups were tested using the t-test for metric data and the Fisher’s test for binary data.

Multiple regression analysis with stepwise forward model selection was performed using remission/complete remission onset (logistic regression) and duration of remission/complete remission (linear regression) as the dependent variables. As covariables, we included age at DM diagnosis, BMI-SDS, HbA1c, pH, HCO_3_^-^, C-peptide, blood lipids (all measured at the time of DM diagnosis), time to remission onset, and insulin daily dose at the discharge from hospital (all as metric data), sex, presence of diabetic ketoacidosis and presence of other autoimmune disease at DM diagnosis (as binary data), and severity of diabetic ketoacidosis (used as a numeric ordinal variable: 0 for no ketoacidosis, 1 for light, 2 for moderate, and 3 for severe DKA). All the covariables were previously tested in the Pearson correlation and univariate regression analyses with a significance level p < 0.2.

P values less than 0.05 were considered as statistically significant. Statistical analyses were performed using SPSSv27 (IBM, NY, USA) and GraphPad Prism 7 (GraphPad, CA, USA) software.

### Ethics committee

The study were approved by the Ethics Committee of the National Institute of Children’s Health in Bratislava, Slovakia, and adhered to the tenets outlined in the Declaration of Helsinki. A written informed consent was obtained from all subjects and/or their legal guardian(s).

## Results

A total of 529 children and adolescents aged 8.5 ± 4.3 years at diabetes diagnosis were included in our study. The basic characterisation of the cohort is displayed in Table 1. Analyses were calculated separately for remission (both partial and complete) and complete remission, and will be presented separately for the sake of clarity.

**Table 1:** Basic characterization of the cohort and comparison between remitters and non-remitters, and partial and complete remitters.

	All	n	No remission	n	Remission	n	p	Complete remission	n	Partial remission	n	p
n (%)	100%	529	60.3%	319	39.7%	210	NA	7.1%	15	92.9%	195	NA
Female	44.4%	235/529	50.8%	162/319	34.8%	73/210	** < 0.001**	40.0%	6/15	34.4%	67/195	0.779
Male	65.6%	294/529	49.2%	157/319	65.2%	137/210		60.0%	9/15	65.6%	128/195	
BMI-SDS	− 0.3 ± 1.12	529	− 0.4 ± 1.03	319	− 0.14 ± 1.23	210	**0.007**	0.16 ± 1.59	15	− 0.16 ± 1.2	195	0.453
Age at DM onset (years)	8.47 ± 4.28	529	7.61 ± 3.98	319	9.77 ± 4.39	210	** < 0.001**	9.67 ± 4.96	15	9.78 ± 4.36	195	0.928
HbA1c (%; mmol/mol)	11.7 ± 2.4; 104 ± 26	529	12.0 ± 2.2; 107 ± 24	319	11.2 ± 2.6; 99 ± 24	210	** < 0.001**	9.5 ± 2.7; 80 ± 30	15	11.3 ± 2.6; 100 ± 28	195	**0.009**
pH	7.35 ± 0.13	529	7.34 ± 0.13	319	7.36 ± 0.12	210	0.079	7.42 ± 0.05	14	7.35 ± 0.13	195	** < 0.001**
HCO3^−^ (mmol/l)	15.65 ± 7.16	529	14.92 ± 7.15	319	16.77 ± 7.04	210	**0.004**	21.15 ± 2.69	14	16.45 ± 7.16	195	** < 0.001**
Total cholesterol (mmol/l)	4.01 ± 0.96	524	4.03 ± 1	316	3.97 ± 0.9	208	0.499	4.38 ± 1.33	14	3.95 ± 0.86	194	0.080
HDL-cholesterol (mmol/l)	1.15 ± 0.36	524	1.14 ± 0.36	316	1.16 ± 0.34	208	0.461	1.11 ± 0.27	14	1.17 ± 0.35	194	0.579
LDL-cholesterol (mmol/l)	2.44 ± 0.82	524	2.45 ± 0.83	316	2.44 ± 0.8	208	0.896	2.77 ± 0.95	14	2.41 ± 0.7	194	0.103
Triglycerides (mmol/l)	0.9 (0.65–1.22)	524	0.9 (0.69–1.21)	316	0.89 (0.61–1.26)	208	0.195*	0.85 (0.71–1.19)	14	0.91 (0.61–1.27)	194	0.475*
C-peptide (pmol/l)	141 (84.5–228)	525	128.5 (76.2–183.5)	316	172 (106–285)	209	< 0.001*	404 (241–462)	15	161.5 (101.7–262.2)	194	< 0.001*
Antibodies (% of positive)	91.9%	463/504	92.4%	279/302	91.1%	184/202	0.621	86.7%	13/15	91.4%	171/187	0.629
Antibodies (% of ≥ 2 positive)	58.3%	295/504	61.9%	187/302	53.5%	108/202	0.065	40.0%	6/15	54.5%	102/187	0.296
IAA (% of positive)	2.2%	11/503	1.7%	5/302	3.0%	6/201	0.360	0.0%	0/15	3.2%	6/186	1.000
IA-2A (% of positive)	69.4%	350/504	71.9%	217/302	65.8%	133/202	0.167	60.0%	9/15	66.3%	124/187	0.778
GADA (% of positive)	80.4%	405/504	82.5%	249/302	77.2%	156/202	0.170	66.7%	10/15	78.1%	146/187	0.339
% with other autoimmunity**	26.7%	144/529	27.6%	88/319	26.7%	56/210	842	20.0%	3/15	27.2%	53/195	0.764
Insulin daily dose at discharge (IU/kg/24 h)	0.4 ± 0.19	529	0.41 ± 0.19	319	0.37 ± 0.19	210	**0.014**	0.29 ± 0.28	15	0.38 ± 0.18	195	0.260
Time from DM diagnosis to remission (days)	NA	NA	NA	NA	123.9 ± 90.18	210	NA	97.07 ± 75.27	15	125.97 ± 91.0	195	0.233
Duration of remission (days)	NA	NA	NA	NA	160.5 (97.5–300.25)	210	NA	279(98–597)	15	154 (96–271)	195	0.030*
Duration of complete remission (days)	NA	NA	NA	NA	NA		NA	294.6 ± 240.42	15	NA	196	NA
% with DKA	37.0%	196/529	40.1%	130/319	31.4%	66/210	**0.034**	6.7%	1/15	33.3%	65/196	**0.041**
HLA risk alleles (% of low/medium/high/very high)	16.0/51.9/11.3/20.8	399	16.9/50.8/9.7/22.5	236	14.7/53.4/13.5/18.4	163	0.497	9.1/36.4/9.1/45.5	11	16.2/52.3/11.3/20.1	152	0.124
T1D genetic risk score (10 SNPs)	12.11 ± 1.41	398	12.04 ± 1.48	235	12.21 ± 1.29	163	0.247	12.47 ± 1.3	11	12.19 ± 1.29	152	0.485

HLA risk alleles for T1D were evaluated as DR3/DR4- very high risk, DR4/DR4 or DR3/- high risk, one DR3 or DR4 allele—medium risk, and none of the risk alleles—low genetic risk for T1D. Metric data are expressed as mean ± SD (normally distributed data) or as median and interquartile range, 25th–75th percentiles (skewed data).

*BMI* body mass index, *SDS* standard deviation score, *DKA* diabetic ketoacidosis, *NA* not applicable, *IAA* autoantibodies against insulin, *IA-2A* autoantibodies against tyrosine phosphatase, *GADA* autoantibodies against glutamic acid decarboxylase.

Significant values are given in bold.

Differences between two groups were tested using t-test for metric data (*Calculated with log transformed data) and by Fisher's test for binary data.

**Among non-remitters had 40 celiac disease, 45 Hashimoto disease, and 3 had both celiac and Hashimoto; in remitters 17 had celiac disease, 34 Hashimoto disease, 1 autoimmune hemolytic anemia, 3 both celiac and Hashimoto, and 1 had Hashimoto and nephrotic syndrome, and in the subgroup of complete remitters 1 had Hashimoto disease, 1 autoimmune hemolytic anemia, and 1 had Hashimoto and nephrotic syndrome. The standard deviation score (SDS) for BMI and height was calculated using local reference values^15^.

### Epidemiology of remission

Remission occurred in 210 (39.7%, CI 35.6–43.9) children and adolescents using the criteria of insulin requirement of < 0.5 IU/kg/day and HbA1c < 7% (Table 1).

### Factors of remission and comparison with non-remitters

While boys and girls were represented nearly equally in the whole cohort, the subset with remission was made up of more than 65% boys (Table 1). In other words, remission was more frequent in boys (137 out of 294) compared with girls (73 out of 235) (46.6%, CI 41.0–52.3% vs 31.1%, CI 25.5–37.2%, p < 0.001).

The remitters compared with the non-remitters had higher age at T1D diagnosis (9.8 ± 4.4 vs 7.6 ± 4.0 years, p < 0.001) (Table 1). Among the age categories, the highest frequency of remission was observed in adolescents aged 15–19 years (71.4%, CI 56.4–82.8%), and the lowest in children aged < 5 years (27.8%, CI 20.9–36.0%). The remitters had higher HCO3^−^ (16.8 ± 7.0 vs 14.9 ± 7.2 mmol/l, 0.004), fasting C-peptide serum levels (172 (106–285) vs 128.5 (76.2–183.5) pmol/l, p < 0.001), and lower HbA1c (11.2 ± 2.6 vs 12.0 ± 2.2%, 99 ± 24 vs 107 ± 24 mmol/mol, p < 0.001) and insulin daily requirements at the time of discharge (0.37 ± 0.19 vs 0.41 ± 0.19 IU/kg/day). Remitters also differed from non-remitters in higher BMI-SDS; however, the mean BMI-SDS values in both groups were in the normal range (− 0.14 ± 1.23 vs − 0.4 ± 1.03, p = 0.007) and the frequency of overweight and obesity was low in both groups (8.6%, CI 5.5–13.1% vs 5.3%, CI 3.3–8.4%). Diabetic ketoacidosis (p = 0.034, OR 0.666, CI 0.465–0.957, Table 1) and severity of diabetic ketoacidosis (p = 0.026, R^2^ = 0.013, β = 0.833, CI 0.711–981 in the logistic regression) were negatively associated with the presence of remission. The presence of other autoimmune diseases and positivity of pancreatic autoantibodies did not have a significant impact on remission occurrence (Table 1). The genetic risk score for T1D and HLA risk alleles were also not associated with remission onset (p = 0.247 for GRS, and p = 0.497 for HLA) (Table 1). In the forward logistic regression, the occurrence of remission was associated with the age at diabetes diagnosis, HbA1c, sex and BMI-SDS (Table 2).

**Table 2:** Logistic regression of remission, and complete remission as dependent variables.

Step	Parameter	ΔR^2^	β (CI)	p value
Dependent variable: remission (R^2^ = 0.175; P < 0.001; n = 520)				
Independent variables: sex, BMI-SDS, age at DM diagnosis, HbA1c, pH, HCO3-, triglycerides*, C-peptide*, insulin dose, DKA				
1	Age at T1D onset	0.080	1.156 (1.102–1.212)	** < 0.001**
2	HbA1c	0.060	0.830 (0.762–0.903)	** < 0.001**
3	Sex (female)	0.021	0.546 (0.372–0.801)	**0.002**
4	BMI-SDS	0.014	1.233 (1.042–1.460)	**0.015**
Dependent variable: complete remission (R^2^ = 0.221; P < 0.001; n = 207)				
Independent variables: HbA1c, pH, HCO3-,C-peptide*, total cholesterol, DKA				
1	C-peptide*	0.221	82.092 (8.085–833.55)	** < 0.001**

Analyzed in forward logistic multiple regression analyses.

*DM* diabetes mellitus, *BMI* body mass index, *SDS* standard deviation score, *DKA* diabetic ketoacidosis.

Significant values are given in bold.

*Calculated with log transformed data.

### Duration of remission

The median of total duration of remission in remitters was 160.5 (IQR 97.5–300.25) days, with the shortest duration lasting 47 days and the longest lasting 950 days. The mean time from diabetes diagnosis to remission onset was 123.9 ± 90.2 days (Table 1). The duration of remission positively correlated with age at DM diagnosis (r = 0.176, p = 0.011) and with higher fasting C-peptide serum levels (r = 0.192, p = 0.005), but not with BMI-SDS (r = 0.129, p = 0.062), HbA1c (r = − 0.099, p = 0.153), pH (r = 0.074, p = 0.288), HCO_3_^−^ (r = 0.086, p = 0.216), total cholesterol (r = 0.018, p = 0.791), HDL-cholesterol (r = 0.010, p = 0.881), triglycerides (r = − 0.011, p = 0.872), lower insulin dose per kilogram at discharge (r = − 0.071, p = 0.308), time to remission onset (r = 0.035, p = 0.610) or genetic risk score for T1D (r = − 0.008, p = 0.916) in Pearson’s correlations. Duration of remission was also not associated with sex (p = 0.398), the presence of diabetic ketoacidosis at DM diagnosis (p = 0.082), other autoimmune diseases (p = 0.853) and positivity for pancreatic antibodies (p = 0.062) in the t-test.

In the forward linear multiple regression analysis, the duration of remission was associated with higher fasting C-peptide serum levels at the time of DM diagnosis (Table 3).

**Table 3:** Multiple forward linear regression of duration of remission, and complete remission as dependent variables.

Step	Parameter	ΔR^2^	Standardized β ± SE	p value
Dependent variable: duration of remission* (R^2^ = 0.037; P = 0.005; n = 202)				
Independent variables: age at DM diagnosis, BMI-SDS, C-peptide*, autoantibodies				
1	C-peptide	0.037	0.192 ± 0.068	**0.005**
Dependent variable: duration of complete remission (R^2^ = 0.572; p = 0.002; n = 14)				
Independent variables: age at DM diagnosis, pH, total cholesterol, C-peptide*, HbA1c, other autoimmune diseases, corticosteroid treatment				
1	corticosteroid treatment	0.572	0.756 ± 0.206	**0.002**

Analyzed in forward linear multiple regression analyses.

*DM* diabetes mellitus, *BMI* body mass index, *SDS* standard deviation score.

Significant values are given in bold.

*Calculated with log transformed data.

### Epidemiology of complete remission

Fifteen participants had complete remission (2.8%, CI 1.7–4.6 from all participants, and 7.1%, CI 4.4–11.4 from all remitters) (Table 1).

### Characterisation of complete remitters

Among the 15 individuals with complete remission were 6 females and 9 males; their age ranged from 1.9 to 17.74 (9.67 ± 4.96) years (Table 1, Table S1). One child had a mild degree of diabetic ketoacidosis, and the remaining 14 children did not have diabetic ketoacidosis at the time of T1D diagnosis. All of them had pH > 7.3. However, 7 participants had HbA1c > 10% (86 mmol/mol). Three children, aged 2, 3 and 4 years, also had other autoimmune diseases (Hashimoto, Hashimoto + nephrotic syndrome, autoimmune haemolytic anaemia). The two children with nephrotic syndrome and autoimmune haemolytic anaemia were treated with corticosteroids at the time of T1D diagnosis. Both of them had ≥ 2 positive pancreatic autoantibodies. After switching corticosteroids to cyclosporine in the boy with nephrotic syndrome and discontinuing corticosteroids in the girl with autoimmune haemolytic anaemia, both had an apparent decrease of insulin dose followed by the complete omitting of insulin treatment for more than 18 months. These 3 children with other diseases also had the longest duration of complete remission (693, 688, and 597 days). For a more detailed characterisation of the complete remitters, see Table S1.

### Factors of the complete remission and comparison to partial remitters

Children with complete remission had a higher pH (7.42 ± 0.05 vs 7.35 ± 0.13, p < 0.001), HCO_3_^−^ (21.2 ± 2.7 vs 16.5 ± 7.2 mmol/l, p < 0.001) and higher fasting C-peptide serum levels (404 (IQR 241–462) vs 161.5 (IQR 101.7–262.2) pmol/l, p < 0.001), as well as lower HbA1c (9.5 ± 2.7 vs 11.3 ± 2.6%, 80 ± 30 vs 100 ± 28 mmol/mol, p = 0.009) at the time of diabetes diagnosis compared with the partial remitters (Table 1). The occurrence of complete remission was not associated with age at DM diagnosis onset (p = 0.928), genetic risk score (p = 0.485) or the HLA risk alleles (p = 0.124) in the t-test; it was also not associated with the severity of diabetic ketoacidosis (p = 0.097, R^2^ = 0.087, β = 0.257) in the logistic regression. In the forward multiple logistic regression, the occurrence of complete remission was only associated with fasting C-peptide serum levels at the time of diabetes diagnosis (Table 2).

### Duration of complete remission

**Figure 1:**
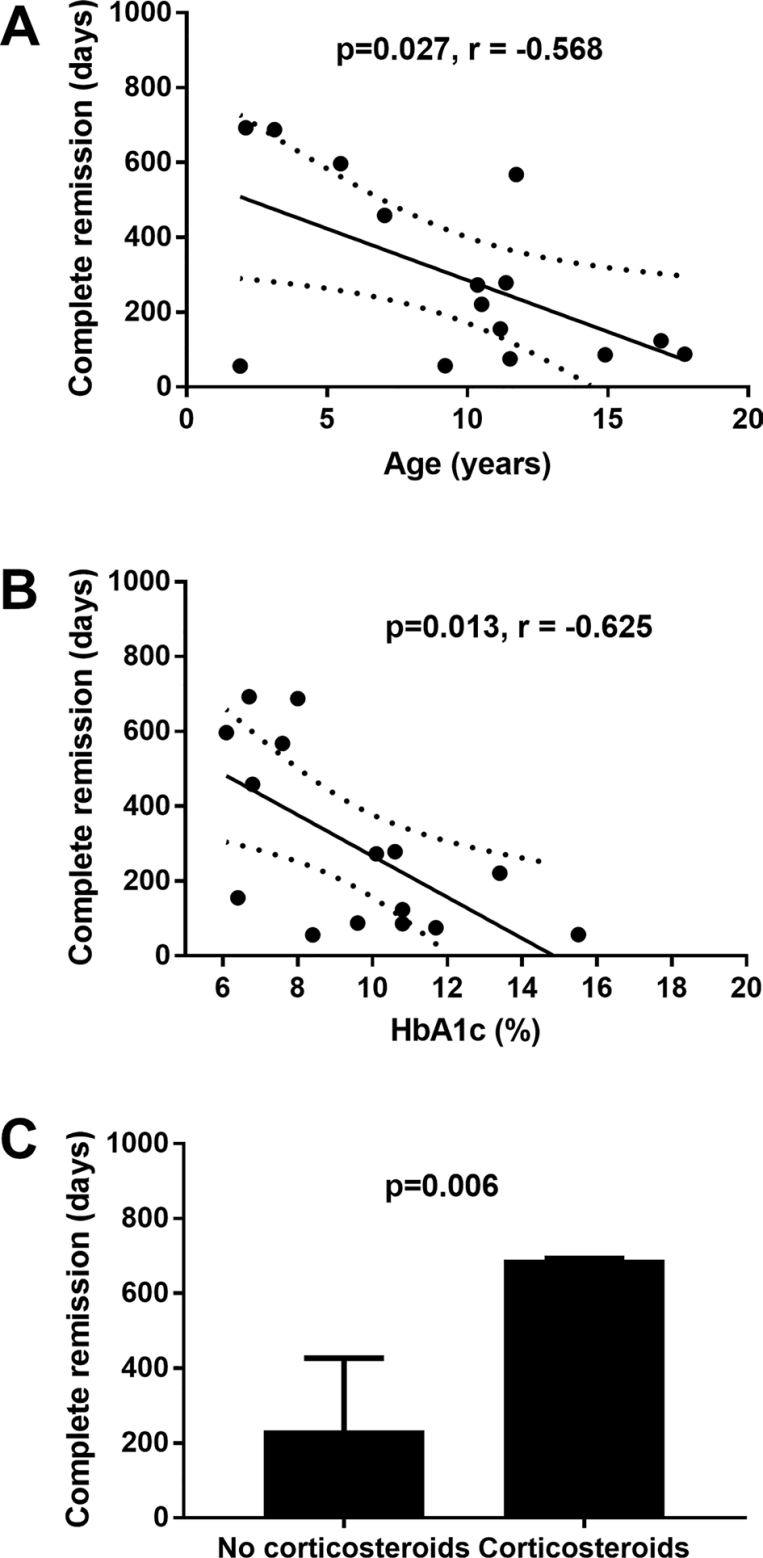
Association of selected factors with the duration of complete remission in children and adolescents with T1D. Pearson’s correlation of the duration of complete remission in complete remitters with age at DM diagnosis (**A**), and HbA1c at the time of DM diagnosis (**B**). Dotted lines represent the 95% confidence intervals of the regression line calculated with the linear regression analysis. Duration of complete remission in complete remitters in the presence of corticosteroid treatment at the time of DM diagnosis (**C**). The error bars in (**C**) show the confidence intervals of the mean. Differences in (**C**) were calculated using the t-test.

The median of total duration of remission was 279 (IQR 98–597) days in complete remitters, and 154 (IQR 96–271) days in partial remitters (p = 0.030) (Table 1). The time without insulin treatment in the complete remission was 294.6 ± 240.4 days (Table 1). The duration of complete remission correlated negatively with age at DM diagnosis (r = − 0.568, p = 0.027) (Fig. 1A) and HbA1c (r = − 0.625, p = 0.013) (Fig. 1B) in Pearson’s correlations and was higher in the t-test in patients also presenting with other autoimmune diseases (i.e. Hashimoto disease, and autoimmune haemolytic anaemia) in the t-test (p = 0.001) or receiving corticosteroid treatment (p = 0.001) (Fig. 1C). The duration of complete remission did not correlate with fasting C-peptide serum levels (r = 0.483, p = 0.068), BMI-SDS (r = 0.206, p = 0.462), pH (r = 0.520, p = 0.057), HCO_3_^−^ (r = − 0.106, p = 0.719), total cholesterol (r = 0.466, p = 0.093), HDL-cholesterol (r = − 0.213, p = 464), triglycerides (r = 0.432, p = 0.123), insulin dose per kilogram at discharge (r = 0.061, p = 0.829), time to remission onset (r = − 0.082, p = 0.771), or genetic risk score (r = 0.270, p = 0.422) in Pearson’s correlations and was not associated with sex (p = 0.725), the presence of diabetic ketoacidosis at DM diagnosis (p = 0.949) or positivity of pancreatic antibodies (p = 0.827) in the t-test. In a forward linear multiple regression analysis the duration of complete remission was associated only with corticosteroid treatment (Table 3).

## Discussion

In our study, remission occurred in 210 (39.7%) children and adolescents with newly diagnosed T1D. Based on the above-presented statistical analyses, we can conclude that the occurrence of remission was higher in older paediatric patients (adolescents) and was more frequent in the boys than the girls. The remission was less frequent in patients with more severe or longer diabetes at disease onset (with higher HbA1c, higher severity of acidosis) and in the patients with lower BMI, respective to their control population (higher negative BMI-SDS). The duration of remission was positively associated with the C-peptide serum levels, which is in line with the logic that remission should depend on the residual capacity of B-cells to produce insulin.

Fifteen participants (2.8%) had complete remission, and these complete remitters had longer duration for the whole remission period compared with partial remitters. The occurrence of complete remission was also associated with the C-peptide levels. Similarly as in the case of remission, the duration of the complete remission was higher in patients with less severe diabetes (lower HbA1c). However, correlation analysis showed that the complete remission lasted longer in complete remitters with a lower age of diabetes onset, which is in contrast with the whole remission group, where the opposite trend was significant. A longer duration of complete remission was associated with corticosteroid treatment as the only independent factor. The factors influencing onset or duration of remission and complete remission in this study are summarised in Table 4.

**Table 4:**
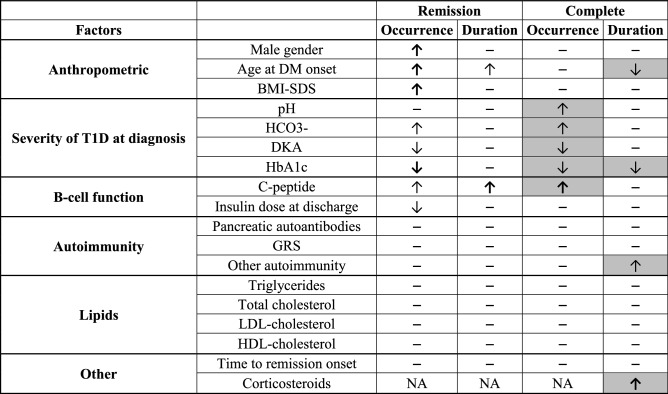
Summary of factors influencing onset or duration of remission and complete remission in this study.

*BMI* body mass index, *SDS* standard deviation score, *DKA* diabetic ketoacidosis, *NA* not analyzed. ↑symbolizes positive association, ↓ symbolizes negative association, arrow in bold symbolizes an independent factor proved in multiple regression analysis, grey field is for not previously reported factor, and —symbolizes no significant association.

### Prevalence and duration of post-initial remission

The prevalence of remission in T1D has been estimated to 35–71% by several studies^7–12,18^. The prevalence of 39.7% in our cohort would therefore belong among the smaller ones reported thus far. There could be several explanations for the differences among studies, including different criteria used, different characteristics of the studied participants or different strategies and targets of the treatment in newly diagnosed T1D^12^.

First, the estimation of the remission prevalence can be influenced by the applied criteria. Large differences in remission prevalence can be seen among studies using the more flexible IDAA1c criteria for remission; Nagl et al.^11^ recorded 71% (Austria & Germany) and Passasini et al.^10^ 63.5% (Italy), Chiavaroli et al.^9^ 42.4% (New Zealand), Marino et al.^8^ 42% (USA), and Nwosu et al.^7^ recorded 35.8% (USA) frequency of remission. However, the ISPAD criteria we used were also used in a Polish study by Chobot et al.^12^, who observed a markedly higher frequency of remission compared with our data (59 vs 39.9%). In our study, the time to remission onset was also longer (2.8 ± 2.5 months^12^ vs 124.0 ± 90.0 days, i.e. 4.1 ± 2.9 months). This may be influenced by different strategies in different centres when following up patients, as well as by the frequency of HbA1c testing. However, the duration of remission was shorter in the Chobot study (7.1 ± 6.6 months vs 240.4 ± 204.0 days, i.e. 7.9 ± 6.7 months)^12^. Therefore, the criteria used most probably did not cause the main difference, although they certainly contribute to the heterogeneity of the studied cohorts.

The participants included in the studies may also differ due to different patient management strategies used, or local dietary habits could even influence the remission occurrence, as was seen in a Czech intervention study, where the prevalence of IDAA1c varied based on the diet (71% on a gluten-free diet, and 35% with no special diet)^18^. In our study, the participants had no special diet, with the exception of children with already confirmed celiac disease.

The prevalence estimation can fluctuate due to the diverse age and gender structure of the cohorts. Several studies have previously reported higher age at DM diagnosis as a factor for remission onset^4,10^, which was also confirmed by this study. The reasons for this could be the higher frequency of diabetic ketoacidosis in younger children and the faster process of B-cell destruction compared with older children and adolescents, both of which are responsible for decreased residual B-cell function at the time of T1D diagnosis^10,19^. In the case of gender, remission was more frequent in boys, similarly to other studies^8,11,20^. One of the explanations could be that girls have a more extensive destruction of B-cells at puberty^21^.

Another factor of remission identified by our study was the severity of diabetes at the onset, as the remitters had significantly lower HbA1c levels at the time of diagnosis. Higher HbA1c levels reflect longer duration and higher values of hyperglycaemia prior to the diagnosis of diabetes and was also described by Wong et al. as a negative factor for remission^22^. In our univariate analyses we also found other factors of remission onset linked to the lower severity of diabetes previously described by others, i.e. higher HCO_3_^−^^8^ and the absence of DKA^9,10^; however, they were not proven to be independent factors in the multiple regression analysis.

Higher C-peptide serum levels were, as in our study, also associated with remission in the study by Passasini et al.^10^. C-peptide reflects the residual function of B-cells at the time of T1D diagnosis. Early diagnosis of T1D is accompanied by higher C-peptide levels and a higher probability of lower insulin daily requirement, which was also seen in our cohort, where the participants with remission had a lower insulin dose at discharge.

The association of remission with BMI-SDS also described in other studies^12^ was sometimes explained by the accelerator hypothesis^23^, in which insulin resistance caused by weight gain worsens glycaemic control and leads to a faster T1D diagnosis. However, the majority of the participants in our study were below or in the normal range for BMI-SDS, and only a few had overweight or obesity (8.6% in remitters and 5.3% in non-remitters). Therefore, it would be more appropriate to talk about lower BMI-SDS due to the later diagnosis of T1D in the non-remission group. This theory of late diagnosis in non-remitters could also be supported by other parameters, e.g. higher HbA1c and lower C-peptide levels in the non-remission group.

Unlike the study by Nagl et al.^11^, we did not find an association between the presence of pancreatic autoantibodies and remission onset. Pancreatic autoantibodies are thought to be associated with more aggressive autoimmune destruction of B-cells^24^, which may also influence remission onset.

We also studied the association of HLA risk alleles and T1D genetic risk score with remission. However, similarly to previous studies associating remission with HLA risk alleles^10^, we did not find any significant associations of the onset of remission with HLA risk alleles. We also did not find any association of onset or duration of the remission with the GRS.

### Prevalence and duration of complete remission

Current knowledge on complete remission is limited. Only a few studies have described some case reports. Our cohort is, to the best of our knowledge, the largest published study on children with complete remission. We found a prevalence of 2.8% in newly diagnosed T1D patients. A study by Abdul-Rasoul estimated the prevalence of spontaneous complete remission at 2.9%^4^. The highest frequency of spontaneous complete remission was recorded by a Canadian study by Martin et al., who described that 25 of 93 participants (27%) did not require insulin treatment for a period of time^5^. However, the onset of complete remission could be influenced by various medicaments and procedures^25^, and today it is hard to find children and adolescents not participating in any clinical trials aimed at the preservation of B-cell function or influencing the spontaneous course of T1D.

Our study is the first to study the factors of onset and duration of complete remission. The only independent factor of the occurrence of complete remission identified among the remitters in our cohort was the C-peptide serum level at the time of DM diagnosis. As mentioned before, a higher concentration of C-peptide signals good condition and secretory capacity for insulin in the B-cells at the time of the T1D diagnosis. In line with this finding, a higher pH and HCO3- and the absence of diabetic ketoacidosis and lower HbA1c at onset, which are also markers of an early diagnosis, were associated with the onset of complete remission in the univariate analysis.

The duration of complete remission was independently associated only with the corticosteroid treatment (used in two participants) at the time of the T1D diagnosis. Corticosteroids, as counter-regulatory hormones, could increase insulin resistance and accelerate the onset of hyperglycaemia despite the good secretory condition of the B-cells and C-peptide serum levels in the normal range (Table S1). This could, in turn, lead to faster T1D manifestation, similarly to the accelerator hypothesis about the effect of higher BMI mentioned above. In the univariate analysis, the presence of other autoimmune diseases also positively correlated with the duration of complete remission. However, in the case of other autoimmunity the association may be skewed by the fact that two of three children with other autoimmune diseases were treated with corticosteroids at the time of their T1D diagnosis. In the univariate analysis, HbA1c at DM diagnosis negatively correlated with the duration of complete remission, thus pointing toward the importance of an early T1D diagnosis.

Interestingly, some factors of complete remission onset and duration differed from the factors identified in all remitters. Boys did not have a higher prevalence of complete remission. Also, age at DM diagnosis was not significantly associated with the onset of complete remission. In fact, there was a negative correlation of age with the duration of complete remission (Fig. 1A). We can only speculate about the explanation for these findings. This association could be influenced by the two young children who were treated with corticosteroids and had long duration of complete remission (Fig. 1A,; Table S1). Without these individuals, the correlation of age with the duration of complete remission would remain negative, though not significant (r = − 0.299, p = 0.321).

### Shortening of remission if insulin treatment is omitted for a period of time

Discontinuation of insulin therapy in complete remission may have several pros and cons. The pros are no hypoglycaemia, no injections, and better quality of life. The cons include false hopes of a possible cure, sometimes worse return to insulin, and also unclear impact on B-cell function. Another question regarding complete remission is whether omitting exogenous insulin for a period of time could shorten the total duration of remission. In our study, total duration of remission was significantly longer in complete remitters compared with partial remitters, which would testify against this hypothesis. However, suspending insulin treatment in complete remitters was not random; thus, we do not know how long the remission would last if they were to be continuously treated with insulin. Therefore, prospective randomised studies will be needed to prove the impact of discontinuing insulin treatment in children with remission.

### Strengths and limitations

The strength of our data is the large cohort of children and adolescents with T1D not participating in any of the clinical trials aimed at preserving B-cell function or influencing the spontaneous course of T1D.

The study has limitations. All patients recruited to this study were diagnosed and treated in one centre, which could influence the generalisation of the results. Moreover, participants were followed-up by four attending paediatric diabetologists who have similar, but not the same, management strategies for T1D. Another limitation is that the ZnT8 autoantibodies were not measured in our study, and the GRS for T1D was not calculated in all the participants. Some analyses in complete remitters could be influenced by the small number of identified children with complete remission.

Another limitation is that there is no consensus and no guidelines (including ISPAD guidelines) regarding the management of T1D remission in children and adolescents^1,26^. There are also no recommendations on how to proceed when complete remission develops, such as whether to discontinue insulin therapy at all or, if so, when to discontinue it and how to monitor the patient after discontinuation. In our study, we used the same approach as in previous studies of complete remission and discontinued insulin treatment for a period of time, during which we monitored glycaemia and reintroduced insulin at the first sign of hyperglycaemia^4,5^. It is also unclear whether such patients can be included in stage 2 or stage 3 intervention trials^26^. Screening programs aimed at detecting early stages of T1D in children and adolescents could provide more information regarding these issues.

### Implications for clinical practice

Most of the identified factors for the onset and duration of partial and especially complete remission point to the importance of early diagnosis of T1D in children and adolescents. Early initiation of treatment may delay fully developed T1D and may be a potential target for intervention studies to protect B-cell function^26^. Our results also underline the importance of T1D screening, which should allow the identification of more children in the early stages of T1D, not only as a prevention of diabetic ketoacidosis^27^, but also to delay manifest T1D by novel biological treatment^28,29^.

## Conclusions

Complete remission is much less frequent in T1D than partial remission. However, both have similar factors influencing their onset and duration. Most of these are related to early diagnosis and better residual pancreatic B-cell secretory function. Quantitative differences in these factors (such as lower HbA1c and higher C-peptide levels) influence whether complete remission, partial remission or no remission occurs. Our results, therefore, highlight the importance of early diagnosis of T1D to achieve not only partial but also to increase the chance of complete remission and to provide a further argument for T1D screening to capture early stages of the disease.

## Supplementary Information

Supplementary Information.

## Data Availability

The datasets used and/or analysed during the current study available from the corresponding author on reasonable request.

## References

[1] Couper JJ (2018). ISPAD Clinical Practice Consensus Guidelines 2018: Stages of type 1 diabetes in children and adolescents. Pediatr. Diabetes.

[2] Steffes MW, Sibley S, Jackson M, Thomas W (2003). Beta-cell function and the development of diabetes-related complications in the diabetes control and complications trial. Diabetes Care.

[3] Mortensen HB (2009). New definition for the partial remission period in children and adolescents with type 1 diabetes. Diabetes Care.

[4] Abdul-Rasoul M, Habib H, Al-Khouly M (2006). 'The honeymoon phase' in children with type 1 diabetes mellitus: Frequency, duration, and influential factors. Pediatr Diabetes.

[5] Martin S (1992). Natural course of remission in IDDM during 1st yr after diagnosis. Diabetes Care.

[6] Hattersley AT (2018). ISPAD Clinical Practice Consensus Guidelines 2018: The diagnosis and management of monogenic diabetes in children and adolescents. Pediatr. Diabetes.

[7] Nwosu BU (2019). Pubertal lipid levels are significantly lower in youth with type 1 diabetes who experienced partial clinical remission. J. Endocr. Soc..

[8] Marino, K. R. *et al.* A predictive model for lack of partial clinical remission in new-onset pediatric type 1 diabetes. *PLoS One* **12**, e0176860. 10.1371/journal.pone.0176860 (2017). 10.1371/journal.pone.0176860 PMC5411061 28459844

[9] Chiavaroli V (2019). Partial remission in type 1 diabetes and associated factors: Analysis based on the insulin dose-adjusted hemoglobin A1c in children and adolescents from a regional diabetes center, Auckland New Zealand. Pediatr. Diabetes.

[10] Passanisi, S. *et al.* Influence of age on partial clinical remission among children with newly diagnosed type 1 diabetes. *Int. J. Environ. Res. Public Health* **17**. 10.3390/ijerph17134801 (2020). 10.3390/ijerph17134801 PMC7369868 32635304

[11] Nagl K (2017). Factors contributing to partial remission in type 1 diabetes: analysis based on the insulin dose-adjusted HbA1c in 3657 children and adolescents from Germany and Austria. Pediatr. Diabetes.

[12] Chobot A (2019). Remission phase in children diagnosed with type 1 diabetes in years 2012 to 2013 in Silesia, Poland: An observational study. Pediatr Diabetes.

[13] Mayer-Davis EJ (2018). ISPAD Clinical Practice Consensus Guidelines 2018: Definition, epidemiology, and classification of diabetes in children and adolescents. Pediatr. Diabetes.

[14] Wolfsdorf JI (2018). ISPAD clinical practice consensus guidelines 2018: Diabetic ketoacidosis and the hyperglycemic hyperosmolar state. Pediatr. Diabetes.

[15] Kobzova J, Vignerova J, Blaha P, Krejcovsky L, Riedlova J (2004). The 6th nationwide anthropological survey of children and adolescents in the Czech Republic in 2001. Cent. Eur. J. Public Health.

[16] Oram RA (2016). A type 1 diabetes genetic risk score can aid discrimination between type 1 and type 2 diabetes in young adults. Diabetes Care.

[17] Locke JM (2020). Methods for quick, accurate and cost-effective determination of the type 1 diabetes genetic risk score (T1D-GRS). Clin. Chem. Lab. Med..

[18] Neuman V (2020). Gluten-free diet in children with recent-onset type 1 diabetes: A 12-month intervention trial. Diabetes Obes. Metab..

[19] Mortensen HB (2010). Multinational study in children and adolescents with newly diagnosed type 1 diabetes: association of age, ketoacidosis, HLA status, and autoantibodies on residual beta-cell function and glycemic control 12 months after diagnosis. Pediatr Diabetes.

[20] Humphreys, A. *et al.* Individual and diabetes presentation characteristics associated with partial remission status in children and adults evaluated up to 12months following diagnosis of type 1 diabetes: An ADDRESS-2 (After Diagnosis Diabetes Research Support System-2) study analysis. *Diabetes Res Clin Pract* **155**, 107789. 10.1016/j.diabres.2019.107789 (2019). 10.1016/j.diabres.2019.107789 31326456

[21] Pozzilli P (2001). Is the process of beta-cell destruction in type 1 diabetes at time of diagnosis more extensive in females than in males?. Eur. J. Endocrinol..

[22] Wong TWC, Wong MYS, But WMB (2021). Features of partial remission in children with type 1 diabetes using the insulin dose-adjusted A1c definition and risk factors associated with nonremission. Ann. Pediatr. Endocrinol. Metab..

[23] Wilkin TJ (2001). The accelerator hypothesis: Weight gain as the missing link between Type I and Type II diabetes. Diabetologia.

[24] Zamaklar M (2002). Relation between course of disease in type 1 diabetes and islet cell antibodies. Ann. N Y Acad. Sci..

[25] Gu W (2012). Diabetic ketoacidosis at diagnosis influences complete remission after treatment with hematopoietic stem cell transplantation in adolescents with type 1 diabetes. Diabetes Care.

[26] Besser REJ (2022). ISPAD Clinical Practice Consensus Guidelines 2022: Stages of type 1 diabetes in children and adolescents. Pediatr. Diabetes.

[27] Ziegler AG (2020). Yield of a public health screening of children for islet autoantibodies in Bavaria Germany. JAMA.

[28] Sims, E. K. *et al.* Teplizumab improves and stabilizes beta cell function in antibody-positive high-risk individuals. *Sci. Transl. Med.* **13**, 1. 10.1126/scitranslmed.abc8980 (2021). 10.1126/scitranslmed.abc8980 PMC8610022 33658358

[29] Herold KC (2019). An anti-CD3 antibody, teplizumab, in relatives at risk for type 1 diabetes. N. Engl. J. Med..

