# Supplementary material for: Complete remission in children and adolescents with type 1 diabetes mellitus—prevalence and factors
Source: Sci Rep. 2023 Apr 26;13:6790. doi: 10.1038/s41598-023-34037-7 (PMC10133219; doi:10.1038/s41598-023-34037-7)
Supplement: Supplementary file 1 — Supplementary Information. [file 41598_2023_34037_MOESM1_ESM.docx]

**SUPPLEMENTAL MATERIAL**

**S-1. Epidemiology of remission using criterion of HbA1c <6.5% (48 mmol/mol)**

Using ISPAD criteria for remission (HbA1c values <7% (53 mmol/mol) and the daily insulin requirement <0.5 IU/kg/day), remission occurred in 210 (39.7%, CI: 35.6–43.9) children and adolescents. Out of them, 110 (52.3%, CI: 45.6-59.0) reached the HbA1c <6.5% (48 mmol/mol) for the median of 201 (IQR 98–343) days.

In the group of complete remitters, 14 children (93.3%, CI: 70.2-99.7) reached the HbA1c <6.5% (48 mmol/mol) for the median of 168 (IQR 85.7–492.2) days. Difference in the duration of remission between the complete and partial remitters using the HbA1c cut-off < 6.5% were not significant (p=0.947).

**S-Table 1. Clinical and laboratory characterization of complete remitters**

| **ID** | **Sex** | **Age at T1D onset** | **BMI SDS** | **Height SDS** | **Celiac or Hash disease** | **Other disease** | **Other treatment** | **HbA1c (%; mmol/mol)** | **pH** | **HCO_3_^-^ (mmol/l)** | **DKA** | **Total choles-terol (mmol/l)** | **HDL-choles-terol (mmol/l)** | **TG (mmol/l)** | **TSH (mU/l)** | **FT4 (pmol/l)** | **C-peptide (pmol/l) (n)** | **Ab** | **Genetic risk score** | **Insulin daily dose at discharge (IU/kg/24h)** | **Duration of complete remission (days)** | **Total duration of remission (days)** |
| --- | --- | --- | --- | --- | --- | --- | --- | --- | --- | --- | --- | --- | --- | --- | --- | --- | --- | --- | --- | --- | --- | --- |
| 1 | male | 14.90 | -1.27 | 0.58 | 0 | 0 | 0 | 10.8; 94 | 7.34 | 18.1 | no | 3.67 | 0.77 | 1.38 | 1.96 | 17.0 | 204 | 1 | 11.5 | 0.32 | 86 | 205 |
| 2 | male | 16.90 | -1.50 | 0.32 | 0 | 0 | 0 | 10.8; 94 | 7.40 | 23.3 | no | 3.29 | 0.92 | 0.78 | 1.54 | 19.0 | 342 | 1 | 12.9 | 0.19 | 124 | 591 |
| 3 | female | 1.90 | 1.11 | 0.51 | 0 | 0 | 0 | 8.4; 68 | 7.44 | 22.5 | no | 4.34 | 1.70 | 0.84 | 2.56 | 17.7 | 462 | 1 | 14.1 | 0.00 | 56 | 56 |
| 4 | male | 11.37 | 2.73 | 0.45 | 0 | 0 | 0 | 10.6; 92 | 7.34 | 14.9 | yes, mild | 4.52 | 1.10 | 1.17 | 1.55 | 21.4 | 196 | 1 | 10.5 | 0.26 | 279 | 279 |
| 5 | male | 17.74 | -1.02 | -0.12 | 0 | 0 | 0 | 9.6; 81 | 7.38 | 23.9 | no | 4.85 | 1.34 | 0.63 | 2.86 | 19.8 | 436 | 1 | 12.1 | 0.48 | 88 | 88 |
| 6 | male | 10.51 | 1.09 | 1.00 | 0 | 0 | 0 | 13.4; 123 | 7.41 | 21.0 | no | 4.39 | 1.63 | 0.6 | 2.77 | 19.8 | 195 | 1 | 13.2 | 0.33 | 221 | 384 |
| 7 | female | 11.52 | 0.03 | 0.66 | 0 | 0 | 0 | 11.7; 104 | 7.43 | 20.8 | no | 3.91 | 1.29 | 0.87 | 2.81 | 19.7 | 321 | 1 | 10.5 | 0.53 | 75 | 75 |
| 8 | female | 7.05 | -0.87 | 0.51 | 0 | 0 | 0 | 6.8; 51 | 7.47 | 23.1 | no | 2.96 | 0.77 | 0.57 | 0.71 | 24.2 | 241 | 0 | 13.2 | 0.00 | 459 | 725 |
| 9 | female | 9.21 | 0.14 | 0.72 | 0 | 0 | 0 | 15.5; 146 | 7.45 | 22.3 | no | 2.50 | 0.85 | 1.27 | 1.71 | 18.2 | 447 | 0 | NA | 0.47 | 57 | 98 |
| 10 | male | 11.17 | -1.28 | 3.05 | 0 | 0 | 0 | 6.4; 46 | 7.40 | 22.9 | no | 4.27 | 1.59 | 1.15 | 4.94 | 12.9 | 448 | 1 | 11.5 | 0.00 | 155 | 155 |
| 11 | male | 11.73 | 3.00 | 1.55 | 0 | 0 | 0 | 7.6; 60 | 7.44 | 24.2 | no | 4.13 | 1.12 | 0.83 | 2.40 | 10.7 | 1034 | 1 | 13.7 | 0.03 | 568 | 568 |
| 12 | male | 10.36 | 0.78 | 1.00 | 0 | 0 | 0 | 10.1; 87 | 7.44 | 19.6 | no | 6.15 | 1.18 | 1.70 | 2.24 | 20.3 | 312 | 1 | 13.8 | 0.22 | 273 | 273 |
| 13 | female | 2.11 | 2.26 | -0.17 | Hash | 0 | L-Th | 6.7; 50 | 7.44 | 21.7 | no | 4.50 | 1.6 | 0.73 | 35.38 | 12.4 | 680 | 1 | NA | 0.00 | 693 | 693 |
| 14 | male | 3.13 | -1.28 | 0.86 | Hash | NS | Cst  L-Th | 8.0; 64 | 7.50 | 17.8 | no | 7.87 | 0.89 | 5.29 | 5.9 | 20.7 | 404 | 1 | NA | 0.54 | 688 | 688 |
| 15 | female | 5.48 | -1.47 | -1.34 | 0 | AIHA | Cst | 6.1; 43 | 7.44 | 22.8 | no | 3.48 | 1.33 | 1.24 | 1.78 | 19.7 | 1034 | 1 | NA | 0.98 | 597 | 597 |

Abbreviations: Hash – Hashimoto disease, DKA - diabetic ketoacidosis, NS – nephrotic syndrome, AIHA – Autoimmune hemolytic anemia, Cst – corticosteroids, L-Th – L-Thyroroxine, TG – triglycerides, Ab – pancreatic autoantibodies: 1 = positive at least one autoantibody, 0 = negative autoantibodies, NA – not analyzed
